# Prevalence of Dementia, Emotional State and Physical Performance among Older Adults in the Metropolitan Area of Guadalajara, Jalisco, Mexico

**DOI:** 10.1155/2014/387528

**Published:** 2014-03-27

**Authors:** Irma E. Velázquez-Brizuela, Genaro G. Ortiz, Lucia Ventura-Castro, Elva D. Árias-Merino, Fermín P. Pacheco-Moisés, Miguel A. Macías-Islas

**Affiliations:** ^1^Centro Universitario de Ciencias de la Salud (CUCS), Universidad de Guadalajara, Sierra Mojada 950, 44350 Guadalajara, JAL, Mexico; ^2^Laboratorio Desarrollo Envejecimiento, Enfermedades Neurodegenerativas Centro de Investigación Biomédica de Occidente (CIBO), Instituto Mexicano del Seguro Social (IMSS), Sierra Mojada 800, 44340 Guadalajara, JAL, Mexico; ^3^Departamento de Salud Pública,Centro Universitario de Ciencias de la Salud (CUCS), Universidad de Guadalajara, Sierra Mojada 950, 44350 Guadalajara, JAL, Mexico; ^4^Departamento de Química, Centro Universitario de Ciencias Exactas e Ingenierías, Universidad de Guadalajara, Blvd. Marcelino García Barragán 1421, 44430 Guadalajara, JAL, Mexico; ^5^Departamento de Neurología, Unidad Médica de Alta Especialidad (UMAE), Centro Médico Nacional de Occidente (CMNO), IMSS, Belisario Domínguez 1000, 44340, Guadalajara, JAL, Mexico

## Abstract

*Background*. Dementia affects memory, thinking, language, judgment, and behavior. Depression, is common in older adults with dementia. The concomitance of dementia and depression increases disability with impaired activities of daily living (ADL), increasing the chances of institutionalization and mortality. *Methods*. Cross-sectional study of a population 60 years and older who live in the State of Jalisco, Mexico. A total of 1142 persons were assessed regarding their cognitive function, emotional state, and physical performance. Door-to-door interview technique was assigned in condition with multistage probability random sampling. Cognitive function, depression and functional disability were assessed by applying standardized Minimental State Examination (Folstein), Geriatric Depression Scale, and the Katz index, respectively. Diagnosis of dementia was performed according to the criteria of the Diagnostic and Statistical Manual of Mental Disorders, the Fourth Edition. Data were analyzed using SPSS software. *Results*. Prevalence of demency was 9.5% (63.35% women, and 36.7% men). Demency was associated with being woman, being older than 70 years, low level of education, not having the economic benefit of retirement, being single or living without a partner, low level of education, suffering from depression and have functional disability in ADL. *Conclusion*. Dementia is more common in women and is related to depression and disability.

## 1. Introduction

We are now facing a demographic transition, where the fertility rate has declined and the life expectancy of individuals has increased; this refers to the decrease in relative and absolute numbers of the younger population, in contrast to an increase in adults aged 60 years and over; this has in turn established an epidemiological transition that entails chronic degenerative diseases, including dementia [1]. Dementia is a clinical syndrome caused by neurodegeneration and is characterized by a progressive decline in brain function manifested by intellectual deterioration [2], especially the capabilities to process (abstract thinking) [3, 4] and to remember new episodic and semantic events [5]. Furthermore, mood disorder, personality, and behavior changes can impinge significantly on social and occupational functioning [6]. In some cases it may be associated with psychotic and motor symptoms. However consciousness remains essentially intact [7, 8].

The nosological classification of dementia includes three main groups: primary degenerative (cortical, subcortical, and cortical-subcortical), vascular, and secondary [9]. Detailed criteria for the diagnosis of dementia are given in the Fourth Edition of the Diagnostic and Statistical Manual of Mental Disorders of the American Psychiatric Association, 1994, the Tenth Revision of the International Classification of Diseases (ICD) [10] and by the National Institute of Neurological and Communicative Disorders and Stroke (NINCDS) and the Alzheimer's Disease and Related Disorders Association (ADRDA) [11]. Recently, the diagnosis of dementia is subsumed under the newly named entity major neurocognitive disorder [12]. However, this proposal is being discussed.

The risk of developing dementia increases with age. Age-standardized prevalence for those aged ≥60 years varied in a narrow band, 5–7% in most world regions, with a higher prevalence in Latin America (8.5%) and Alzheimer's disease accounts for 50 to 80 percent of dementia cases [13] and affects approximately 10% of people over 65 and 50% of people over 85 years. Vascular dementia (VaD) is the second most common form of dementia [7].

Depression and dementia are closely related. However, the relationship is complex and the mechanism linking remains unknown; depression can coexist with dementia or can also be confused with dementia, hence, the importance of a differential diagnosis [14]. Furthermore, it was found that dementia affects performance in everyday life to produce total disability [15].

To date there are 4.86 million people older than 60 years of age living in Mexico. In 1970, the percentage of adults 65 years and older accounted for 4% of the total population. It is estimated that in 2050 there will be 35 million elderly people [16]. Therefore, a significant increase in the geriatric population is expected in Mexico during the next decades.

The worldwide prevalence of dementia is 6.2% (8.8% women, 3.1% men). That figure is projected to almost the double every 20 years, reaching 65.7 million by 2030 and 115.4 million by 2050. The majority of these people will be living in low- and middle-income countries [17]. Currently, there are over 4.5 million people with dementia in the USA and only 25% have been properly diagnosed and 10% are adequately treated. A study of Mexican and Central American Hispanics in California found prevalence of 4.8% in those aged 60–69 years and 31% in those over 85 years [18]. It has been reported the existence of 500,000–700,000 patients with dementia in México; however, less than 25% have been properly diagnosed. Furthermore, primary care and other specialists often lack knowledge or skill for appropriate screening, diagnosis and treatment of dementia [19].

The population-based study conducted in Mexico for the assessment of dementia in the elderly shows prevalence rates of 7.4 and 7.3% for urban and rural localities, respectively [20], while another study in México City shows a prevalence of 6.1% [21]. Reported prevalence estimates for Tepatitlan, JAL, Mexico,are about 0.33% in elderly subjects, and women were the most affected [22]. A similar study done at family medicine units of the Mexican Social Security Institute (IMSS, Instituto Mexicano del Seguro Social) reported that prevalence of dementia was 3.5% in people over 60 years and higher prevalence in women. Data from the Mexican Health and Aging Study project showed prevalence rates of 6.1% for dementia and higher estimates in women only in the oldest subjects [21].

Dementia is a public health problem, which is mainly characterized by a decrease of memory in intellectual abilities, which alters the operation of the activities of daily living. This decrease should be objectively shown through a detailed medical history, laboratory tests and cabinet, and a neuropsychological evaluation. From the clinical point of view, it is important to make a differential diagnosis of the causes and specific diseases causing dementia, as this has direct implications for prognosis and treatment [23].

The trend toward longer life has also raised concerns about the quality of life at older ages. Therefore, it is necessary to take effective preventive and public health measures for the diagnosis and early treatment of the older adults with dementia in order to regain their physical health and improve their performance of daily activities. The aim of the current study was to determine the prevalence of dementia and to evaluate the physical function and emotional status of adults aged 60 years and over in the metropolitan area of Guadalajara, JAL, Mexico.

## 2. Materials and Methods

A descriptive cross-sectional study was conducted door to door. The study was conducted in the metropolitan area of Guadalajara (GMA), México, which includes the city of Guadalajara and its surrounding municipalities: El Salto, Tlajomulco, Tlaquepaque, Tonala, and Zapopan. The six municipalities of GMA are subdivided into 14 urban basic geostatistical areas (UGEA). Multistage and proportional random sampling was used to obtain our study sample of adults. The sample size obtained was 1142 adults, 60 years of age and older. Within the 14 UGEAs, we conducted the random selection of blocks. Locating the block, we proceed at the southwest corner clockwise until we find an adult 60 years or more. Adults aged 60 years or more living at least one year at the GAM were invited to participate. Data were collected by means of structured personal interviews conducted by trained interviewers. All participants or family members provided informed consent.

In the first stage, sociodemographic data (age, gender, marital status, education, and occupation) were obtained from the first interview. Cognitive impairment was measured using the Spanish version of the Minimental State Examination [24]; it provides a brief evaluation of the cognitive domains including orientation, registration, attention, recall, language, and constructional praxis. Patients' scores range from 0 to 30, with low scores indicating greater cognitive impairment. Cutoffs were used to grade severity of change as mild, moderate, or severe. Namely, mild cognitive impairment was considered when the patient was rated 20–24 points, moderate cognitive impairment with 16–19 points, and severe cognitive impairment with 15 or fewer points. The cutoff score for dementia is 19 points. The test sensitivity was 87%, and specificity was 82%.

The second stage of the study was done as soon as older adults were identified with moderate-to-severe cognitive impairment (dementia). Depression was assessed using the Spanish version of the Geriatric Depression Scale (GDS) of 30 items [25]; a cutoff point of 10/11 was considered for depression and nondepression. Functional disability was measured in terms of limitations in ADL using the Katz Index [26]; the items included were bathing, transferring, dressing, toileting, continence, and feeding; response categories provided were “independent” (if person needs no help or minimal help to perform the activity) or “dependent” (if person needs assistance to perform the activity); not being able to perform at least one of the tasks unassisted was considered a dependence in ADL and a cutoff point was assigned of 5/6 dependent/independent on a dichotomous scale.

Diagnosis of dementia was performed according to the criteria of the Diagnostic and Statistical Manual of Mental Disorders, the Fourth Edition (DSM-IV). We performed the statistical analysis with the Statistical Package for the Social Sciences (SPSS) v16.0 package for Windows. The prevalence of dementia, the degree of functional dependence, and depression were calculated as a percentage. Chi-square test (*P* < 0.05) was used to analyze differences between proportions of men and women for each independent variable. Odds ratio (OR) and their associated 95% confidence intervals (95% CI) were computed using descriptive statistics.

This research took into account the Regulation of the General Law of Health in health research and ethical aspects of human research (Mexico); this was carried out in accordance with the principles of the Declaration of Helsinki. For the type of approach, this study is considered as the minimal risk. The letter informed consent was developed by the investigators. The study was approved by the National Research and Ethics Committee and research of the Mexican Social Security Institute (IMSS).

## 3. Results

In the first stage we were able to obtain complete sociodemographic data from 1142 elderly aged above 60 (413 men, 729 women). Mean age of participants was 71.6 ± 8.3 years, ranging from 60 to 110 years of age. Cognitive function was assessed by applying standardized Minimental State Examination at all participants.

As shown in Table 1, the overall prevalence of cognitive impairment in the sample was 13.8% (158). Mild cognitive impairment was found in 4.3% of subjects (49). 109 subjects (9.5%) have dementia; namely, 6.9% (79) and 2.6% (30) were suffering from moderate and severe cognitive impairment, respectively.

Sociodemographic data showed that women showed a higher proportion of dementia than men (63.3%, 69 versus 36.7%, 40) and significant differences by gender (*P* = 0.001) were found. The schooling range was 0 to 23 years, and the average was 4.12 years (3.62 for women and 4.67 for men). Significant differences were observed in education years by gender (*P* = 0.030). 21.1% (23 subjects) had no formal educational instruction. Regarding marital status, 55.5% were married or living with a partner and 44.5% were single or living without a partner; significant differences by gender were found (*P* = 0.020). 82.5% (90 subjects) of the sample did not have the benefits of retirement or have a pension, while 17.5% are retirees and pensioners (6.4% women and 11.1% men). Significant differences in the benefit of retirement or pension between men and women were found (*P* = 0.002). Women showed a higher proportion of depression than men (22.0% and 10.1%, resp.) with significant differences by gender being found (*P* = 0.001).

Data analysis of odds ratio showed in Table 2 suggests that the prevalence of dementia is associated with being women (OR = 14.2; IC 95%, 12.20–17.41), being older than 70 years (OR = 47.7; IC 95%, 39.70–56.91), low educational level (OR = 21.2; IC 95%, 12.20–32.40), not having the benefits of retirement (OR = 11.2; IC 95%, 9.20–19.40), being single or living without a partner (OR = 5.41; IC 95%, 4.79–7.87), having depression (OR = 2.56; IC 95%, 2.31–4.80), and having functional disability in activities of daily living (OR = 6.05; IC 95%, 3.4–9.6).

Data in Table 3 shows that depression in patients with dementia is associated with being women (OR = 2.56; IC 95%, 2.31–4.80), being older than 75 years (OR = 1.76; IC 95%, 0.98–3.01), being single or living without a partner (OR = 1.93; CI 95%, 0.75–3.16), not having the benefits of retirement (OR = 1.90 CI 95% 1.01–3.23), and low educational level (less than four years) (OR = 2.89; CI 95%, 1.35–4.56).

The prevalence of functional impairment among subjects aged 60 years and above was 71.55% (78 persons). Among the aspects of activities of daily living which were being assessed, the common disabilities were inability to bath or shower followed by bladder or bowel control (significantly higher in women than in men, *P* = 0.000), dressing, and eating.

Table 4 shows the relation of sociodemographic variables and health characteristics in activities of daily living which showed that being a woman is a condition that is more likely to have dependency for activities of daily living (OR = 6.05; IC 95%, 3.4–9.6) than being a man. Age was another factor associated with people older than 75 years, in comparison to elderly people that were younger (OR = 4.86; IC 95%, 3.22–6.58). Elderly people without a partner had a higher probability of being dependent (OR = 2.54, IC 95%, 1.92–3.36) than those who were married or had a partner, and also having a low education level (less than or equal to 4 years of studies) (OR = 1.50; IC 95%, 1.11–2.03). Not having a pension was also related to disability in activities of daily living (OR = 1.88; IC 95%, 1.64–1.20). Dementia and depression in elderly individuals were associated with disability in activities of daily living (OR 16.41; IC 95%, 14.80–19.61).

## 4. Discussion 

The data obtained in this study show that the overall prevalence of dementia in a representative sample of elderly adults of the metropolitan area of Guadalajara, JAL, was 9.5%, being higher in women (6.0%) than in men (3.5%). The overall prevalence found in this work is slightly higher than the reported in a population-based study carried out for the evaluation of dementia in Mexico, where it indicates that the prevalence of dementia for middle and lower-middle income older adults was 8.6 for urban areas [7]. A study conducted in four family medicine clinics of the Mexican Institute of Social Security (IMSS) located in Guadalupe, Nuevo León, showed that the prevalence of dementia was 3.5% in adults, from 60 years and older (1.2% men, 2.3% women; *P* < 0.015) [19], although these data must be taken with caution, because the sample covers only part of the population that has the privilege of social security. Another study done at the Mexican state of Jalisco, specifically in the City of Tepatitlan, reported a prevalence of dementia of 3.3 in 1000 with a slight female predominance over 60 years old [22].

A number of studies have found that elderly women were much more likely to have dementia than men and that the likelihood of having dementia kept increasing with age for the women but not for the men. However, it is worth considering that more women than men survive at the oldest ages. Interestingly, 4.3% of older adults suffer from mild cognitive impairments that will largely increase their risk of developing dementia. The term mild cognitive impairment defines a heterogeneous group of individuals who are at a risk greater than that observed in the general population for the development of dementia, particularly Alzheimer's disease [27]. Identifying the risk factors of cognitive impairment in our population is essential for developing interventions that could prevent or delay the onset of dementia. Patients suffering from cognitive impairments usually have a poor prognosis, increased risk of institutionalization, and are a major cause of stress for caregivers. For this reason, early detection of cognitive impairment is beneficial for the patient, family, and health sector.

The prevalence of depression found in this study was 32.1% and was higher in women (22.0%) compared to men (10.1%) (OR = 2.56; 95% CI, 2.31–4.80). Furthermore, depression was associated with cognitive impairment. These data are consistent with a previous study [14]. Our results suggest that dementia was associated with depression (OR = 2.56; IC 95%, 2.31–4.80). In addition a strong relationship between depression and cognitive functioning among older Mexican Americans was reported (OR = 3.75; 95% CI, 2.63 to 5.34) [28].

Results showed that depression in patients with dementia was significantly associated with the sociodemographic data analyzed (being female, being over 75, being single or living without a partner, not having the benefits of retirement, and a low educational level). These data coincide with the studies in Jalisco and México where the prevalence of cognitive impairment and depression was reported previously [29, 30], suggesting that prevalence of both conditions has not changed in the last few years.

Although it might be especially difficult to differentiate depression from dementia in older adults we also know that 30% of patients with Alzheimer's dementia present, initially, with depressive features, and, in general, when depression affects adults older, gives poor prognosis, since 50% of them will develop in the next 2 years true dementia symptoms [31]. We agree with Blazer and Williams (1980) on the need to take into account the difficulty of diagnosing depression in older people with dementia [32]. This represents a major threat to the epidemiological investigation, since many of the symptoms of the person experiencing depression can be attributed to the aging process, to disturbances in the central nervous system, or to dementia itself.

Analysis of data regarding ability to perform activities of daily life and dementia showed that 71.55% of subjects were dependent. In addition, being women is a strong risk factor for the association of ADL and disability. These findings are similar to the reported ones by Wray and Blaum [33], who found that gender has a direct effect on disability through depressive symptoms [33]. Bruce [34] supports this hypothesis, indicating that depression and disability are mutually reinforcing. Furthermore, in this study, people over age 60 were more dependent in activities of daily life, compared to younger people. It is worth noting that limitations in cognition and inability to perform activities of daily life are inherent to dementia and essential components of its severity. Examination of this disability is crucial for diagnosis, management of the patient and family, and evaluation of treatment effects.

Being an older adult single or living without a partner is a condition that has a higher probability of having difficulties in activities of daily life. Other variables associated were a low educational level and not having the benefits of a pension. These results are consistent with the reported previously, regarding age [35–40], marital status [38], low educational level [39, 41], cognitive impairment [40], and depression [42].

Dementia in geriatric patients is frequently underdiagnosed in primary care. The objective of this study was to identify its prevalence in geriatric patients in the metropolitan area of Guadalajara. The way the population is distributed over 60 years of age in the study showed a universal behavior, as most were located in the first five-year periods from that age, and these population groups were attending the most attention to public sector hospitals. The results exhibited in this paper were consistent with those reported worldwide.

The previous study draws attention to the high percentage of patientswith mild cognitive impairment, since being a chronic dementia raises the question of how many are going to progress in later years to moderate or severe cognitive impairment and dementia.

## 5. Conclusions 

Dementia is a major cause of disability and dependency among older adults and can be overwhelming not only for sufferers but also their caregivers and families. There are significant issues in terms of the direct costs of medical, social, and informal care associated with dementia. Moreover, physical, emotional, and economic pressures can cause great stress to families. Support is needed from the health, social, financial, and legal systems for both people with dementia and their caregivers.

Data reported in this paper are exclusively for a metropolitan area of Guadalajara, México. The number of available epidemiological studies in Mexico was still too small to meet more accurately the situation of dementias. Therefore, it is desirable to have a National Survey of Dementia or include in the National Survey on Aging information relative to dementia that allows us to know with certainty the situation of dementias in our country. Today the priority is to identify the risk factors and the cognitive impairment beginning in our population in relation to dementia care to achieve comprehensive care and education to the patient, the caregiver, and family.

## Figures and Tables

**Table 1:** Mental status of participants according to age and sex of f participants included in this study (*n* = 1142 older adults).

	Without cognitive impairment f (%)	Mild cognitive impairment f (%)	Demency f (%)	Total f (%)
Age groups (years)				
60–69				
Women	258 (22.59)	13 (1.13)	12 (1.05)	283 (24.78)
Men	191 (16.72)	6 (0.52)	12 (1.05)	209 (18.30)
70–79				
Women	167 (14.62)	9 (0.78)	20 (1.75)	196 (17.16)
Men	88 (7.70)	5 (0.43)	16 (1.40)	109 (9.54)
80–89				
Women	167 (14.62)	9 (0.78)	27 (2.36)	203 (17.77)
Men	68 (5.95)	3 (0.26)	10 (0.87)	81 (7.09)
≥90				
Women	27 (2.36)	3 (0.26)	10 (0.87)	40 (3.50)
Men	18 (1.57)	1 (0.08)	2 (0.16)	21 (1.83)

Total	984 (86.16)	49 (4.3)	109 (9.5)	1142 (100)

**Table 2:** Association between sociodemographic characteristics and mental and physical status (*n* = 109 older adults with dementia).

Variable	Mujeres f (%)	Hombres f (%)	Total f (%	OR	
Gender					
Women	69 (63.3)	—	69 (63.3)	14.2	2.20–17.41
Men	—	40 (36.7)	40 (36.7)		
Age groups (years). Mean = 71.6, standard deviation (SD) = 8.3					
60–69	12 (11.0)	12 (11.0)	24 (22.0)	47.7	39.70–56.91
70–79	20 (18.35)	16 (14.67)	36 (33.02)		
80–89	27 (24.77)	10 (9.17)	37 (33.94)		
90–99	8 (7.33)	2 (1.83)	10 (9.17)		
100–110	2 (1.83)	0 (0.0)	2 (1.83)		
Education (years). Mean = 4.12 (3.62 for women and 4.67 for men) (*P* = 0.070).					
	13 (11.92)	10 (9.17)	23 (21.10)	21.2	12.20–32.40
1–4	39 (35.77)	21 (19.26)	60 (55.04)		
5-6	15 (13.76)	8 (7.33)	23 (21.10)		
7>	2 (1.83)	1 (0.91)	3 (2.75)		
Marital status					
Married or free union	38 (34.86)	27 (24.77)	65 (55.5)	5.41	4.79–7.87
Single, widowed, or divorced	31 (28.44)	13 (11.92)	44 (44.5)		
Retired status					
Pension	7 (6.4)	12 (11.1)	19 (17.5)	11.2	9.20–19.40
No pension	62 (56.88)	28 (25.68)	90 (82.5)		
Depression					
Yes	24 (22.0)	11 (10.1)	35 (32.1)	2.56	2.31–4.80
Not	45 (88.0)	29 (89.9)	74 (67.9)		
Dependency for activities of daily living					
Yes	59 (85.50)	19 (47.50)	78 (71.55)	6.05	3.4–9.6
Not	10 (14.50)	21 (52.50)	31 (28.45)		

**Table 3:** Association between sociodemographic characteristics and depression (*n* = 109 older adults with dementia).

Variable	With depression f (%)	Without depression f (%)	OR	CI (95%)
Gender				
Woman	24 (34.7)	45 (65.3)	2.56	2.31–4.80
Man	11 (27.5)	29 (72.5)	1	
Age group (years)				
60–75	5 (4.58)	28 (25.68)	1	0.98–3.01
≥75	30 (27.52)	11 (10.09)	1.76	
Marital status				
Single, widowed, or divorced	30 (27.52)	16 (14.67)	1.93	0.75–3.16
Married or free union	5 (4.58)	23 (21.10)	1	
Retired status				
No pension	28 (25.68)	14 (12.84)	1.90	1.01–3.23
Pension	7 (6.42)	25 (22.9 3)	1	
Education (years).				
0–4	27 (24.77)	15 (13.76)	2.89	1.35–4.56
≥5	8 (7.33)	24 (22.01)	1	

**Table 4:** Association between sociodemographic characteristics and dependency for activities of daily living (*n* = 109 older adults with dementia).

Variable	Dependent, *n* = 78 f (%)	Independent, *n* = 31 f (%)	OR	CI (95%)
Gender				
Woman	59 (85.50)	10 (14.50)	6.05	3.4–9.6
Man	19 (47.50)	21 (52.50)	1	
Age group (years)				
60–69	9 (8.25)	15 (13.76)	1	
≥70	69 (63.30)	16 (14.67)	4.86	3.22–6.58
Marital status				
Single, widowed, or divorced	34 (31.19)	10 (9.17)	2.54	1.92–3.36
Married or free union	21 (19.26)	44 (40.36)	1	
Education (years).				
0–4	63 (57.79)	20 (18.34)	1.50	1.11–2.03
≥5	15 (13.76)	11 (10.09)	1	
Retired status				
No pension	70 (64.22)	20 (18.34)	1.88	1.64–1.20
Pension	8 (7.33)	11 (10.09)	1	
